# Kidney-Targeted Gene Transfer: Therapeutic Advances in Native Kidney Disease and Emerging Ex Vivo Allograft Engineering

**DOI:** 10.3390/genes17101270

**Published:** 2026-10-13

**Authors:** Yongfa Lei, Ruying Zheng, Daqiang Zhao

**Affiliations:** 1 Institute of Organ Transplantation, Tongji Hospital, Tongji Medical College, Huazhong University of Science and Technology, Wuhan 430030, China; 2 Key Laboratory of Organ Transplantation, Ministry of Education, Wuhan 430030, China; 3 National Health Commission Key Laboratory of Organ Transplantation, Wuhan 430030, China; 4 Key Laboratory of Organ Transplantation, Chinese Academy of Medical Sciences, Wuhan 430030, China; 5 Hubei Provincial Clinical Research Center for Organ Transplantation, Wuhan 430030, China

**Keywords:** kidney gene transfer, gene therapy, adeno-associated virus, genome editing, ex vivo machine perfusion, kidney transplantation

## Abstract

Kidney-targeted gene transfer is progressing toward two distinct clinical applications: correcting molecular defects or modifying disease pathways in native kidneys and modifying donor kidneys before allotransplantation to reduce ischemia–reperfusion injury or rejection. These applications share a delivery challenge but differ in their target cells, therapeutic mechanisms, required duration and acceptable systemic exposure. Advances in renal delivery have produced disease benefit in selected animal models, while ex vivo perfusion offers a controlled opportunity to expose and assess a graft before implantation. Neither renal vector detection nor reporter expression alone establishes therapeutic efficacy. This review evaluates the evidence through four linked layers: vector distribution, cell-resolved delivery or expression, therapeutic target engagement and functional benefit. It compares candidate strategies according to indication and duration, distinguishes capsid tropism from promoter-mediated expression bias and separates demonstrated outcomes from mechanistic proposals. For donor-kidney engineering, limited treatment time, uneven intrarenal delivery, perfusion injury and delayed vector release after reperfusion remain central constraints. Translation will require indication-specific dose–response studies, human-relevant delivery assessment and surveillance extending beyond early benefit or transient immune silencing. Progress therefore depends on a coherent therapeutic configuration, rather than a universal kidney vector.

## 1. Introduction

Gene therapy offers a way to address defined molecular defects and modify disease pathways, yet the kidney remains a difficult delivery target [1]. Chronic kidney disease (CKD) encompasses diverse etiologies and anatomical compartments [2]. Genetic diagnoses identify molecular defects in podocytes and tubular epithelia [3,4], while acquired glomerular inflammation and kidney-allograft injury introduce different disease mechanisms. Restoring a structural protein, secreting an immunomodulatory enzyme and transiently suppressing antigen presentation consequently require different delivery and safety strategies.

Two clinical paths organize this review. In native kidneys, treatment must reach the cells responsible for a defined disorder and produce sufficient correction without unacceptable systemic exposure. In transplantation, the donor kidney can be treated before implantation, creating an opportunity to modify ischemia–reperfusion injury (IRI) or alloimmune recognition while controlling the initial exposure. Recent work includes podocin replacement and context-dependent or engineered adeno-associated virus (AAV) delivery [5,6,7,8], cassette optimization in murine and human renal tissue [9], endothelial immunomodulation and sequence correction [10,11] and perfusion-mediated RNA interference [12,13,14]. These findings support different stages of development rather than equivalent readiness for clinical use.

This narrative review examines representative renal gene-transfer studies and their translational implications. It distinguishes nonspecific renal signal—bulk detection without identifying the relevant cell or demonstrating biological activity—from cell-resolved delivery and therapeutic benefit. The discussion relates the demonstrated evidence to disease mechanism, therapeutic configuration and the validation needed for clinical development.

## 2. Biological Barriers and Translational Determinants of Kidney Gene Transfer

**Figure 1:**
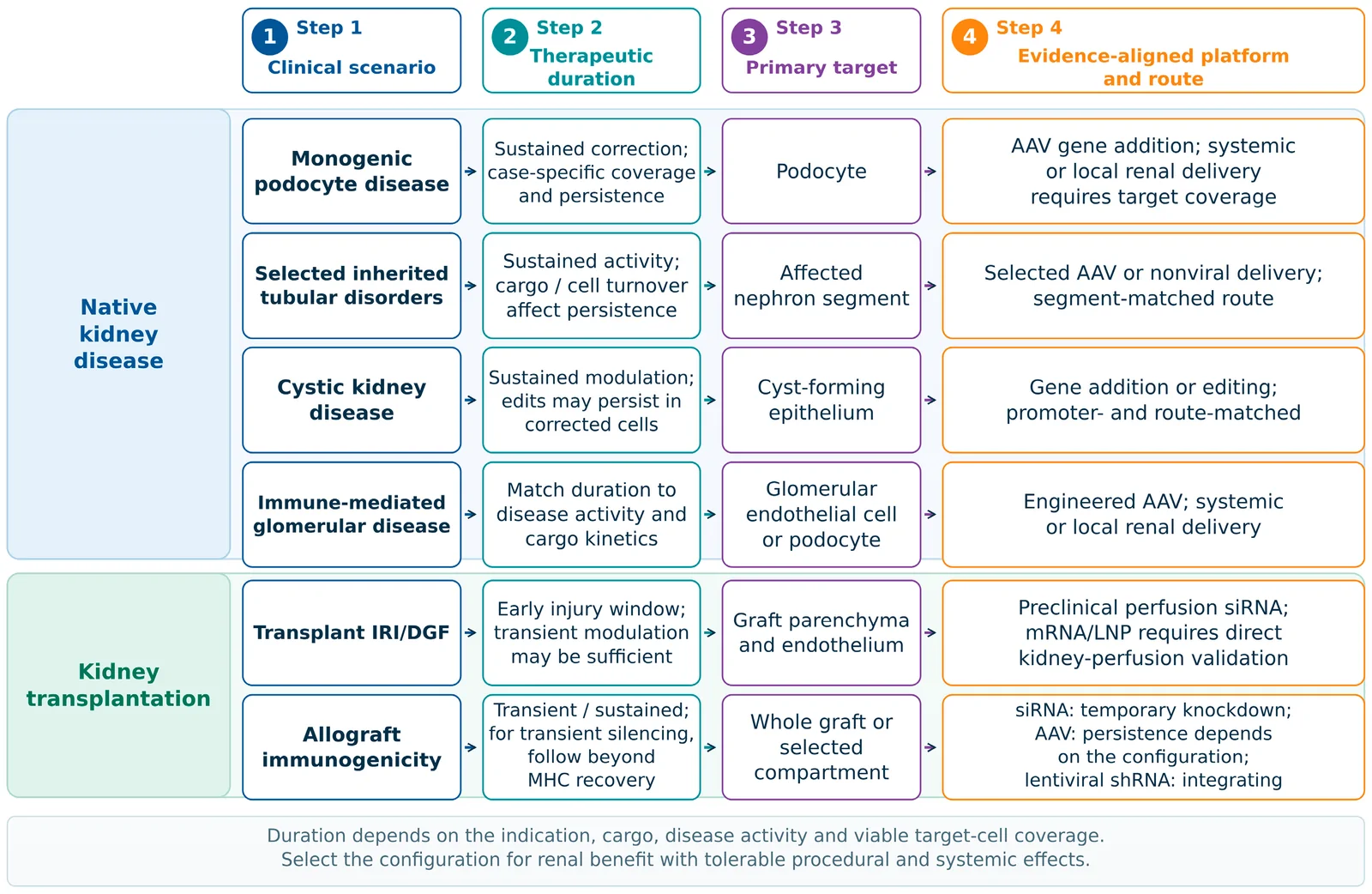
**Indication- and target-compartment-led framework for kidney-targeted genetic therapeutics.** The indication guides the desired duration, target compartment, platform and route. Duration depends on disease activity, cargo pharmacology, cell turnover, viable target-cell coverage and endpoint; it is not assigned uniformly by disease category. Persistent expression and permanent correction of an edited cell are distinct. Listed configurations include experimentally supported and candidate approaches whose evidence cannot be transferred across species, diseases or routes. Perfusion-delivered siRNA has renal preclinical support; donor-kidney mRNA/LNP treatment requires direct validation. Follow transient MHC modulation beyond measured MHC recovery. Renal promoter bias does not establish kidney-exclusive vector biodistribution; configuration selection requires renal benefit with tolerable procedural and systemic effects. Abbreviations: AAV, adeno-associated virus; DGF, delayed graft function; IRI, ischemia–reperfusion injury; LNP, lipid nanoparticle; mRNA, messenger RNA; MHC, major histocompatibility complex; shRNA, short hairpin RNA; siRNA, small interfering RNA.

Therapeutic design begins with the disease indication and the renal compartment that must be reached. Figure 1 summarizes how these choices guide the required duration of activity, delivery platform and administration route for native-kidney treatment and donor-kidney engineering. Each configuration must be evaluated for renal benefit and tolerable procedural and systemic effects.

### 2.1. Operational Definition and Levels of Evidence

We use “kidney-targeted” to describe a delivery configuration designed to achieve activity in a specified renal compartment and supported by evidence of renal delivery or activity. The strength of that claim depends on cell-resolved measurements, an appropriate comparator and assessment of extra-renal exposure in relation to benefit and tolerability. It does not imply kidney-exclusive biodistribution: renal promoter bias can coexist with liver exposure, and a protein produced in the kidney can enter the circulation [5,10,11].

Four evidence layers should be reported separately. Distribution concerns vector or cargo in renal and extra-renal compartments. Cell-resolved delivery or expression identifies the exposed or expressing cell population. Target engagement demonstrates the intended molecular action, such as restored podocin localization, reduced major histocompatibility complex (MHC) expression or sequence correction. Functional efficacy requires a disease-relevant benefit, such as reduced albuminuria, preserved filtration or improved graft survival. Whole-kidney vector-genome quantitative polymerase chain reaction (qPCR), homogenate fluorescence and bulk tissue staining are examples of organ-level signals: they can include residual intravascular material, uptake without expression or signal from unintended cells. These measurements cannot substitute for the later evidence layers.

The evidence category assigned here is the highest directly demonstrated outcome in the cited experimental configuration. Reporter or molecular proof-of-concept establishes delivery, expression or target engagement. Therapeutic preclinical evidence additionally requires benefit in a disease or transplant model. Clinical-development evidence denotes a formally described human study, without assuming efficacy. We reserve translational maturity for a configuration with reproducible human benefit, an acceptable safety profile and a practicable treatment process. Evidence in one species, cargo or delivery route does not automatically raise another configuration to the same category.

### 2.2. Dynamic Renal Barriers and Specificity

Renal access depends on vascular anatomy, the glomerular filtration barrier, epithelial polarity and clearance. Passage from glomerular capillary blood to urinary space requires traversal of the endothelial surface, basement membrane and podocyte-associated barrier. In active proteinuric disease, slit-diaphragm disruption and basement-membrane injury could alter this passage, but individual lesions have not been shown to produce a predictable increase in AAV permeability [15]. Foot-process effacement alone is not evidence of viral passage. Podocyte detachment could increase local exposure while reducing the number of viable cells available for transduction; consequently, more severe injury need not yield more useful gene transfer. These are mechanistic hypotheses to test in each disease, rather than a general property established for all active glomerulopathies.

Furusho et al. provided direct evidence that disease context changes vector handling: after intravenous administration in *Col4a5*-mutant Alport mice, more than 1% of the administered AAV9 genome dose was recovered in urine over 5 h, compared with approximately 0.001% in healthy controls; the urinary increase for AAV-KP1 was not statistically significant [6]. This supports increased passage of vector-derived material into urine, but qPCR does not identify intact infectious particles, and urinary recovery does not demonstrate productive uptake by podocytes or tubular cells. Cell-resolved expression must therefore be assessed separately from urinary vector genomes and proteinuria. Separate reporter analyses showed enhanced AAV9 transduction of podocytes and proximal tubules in the Alport mice; AAV-KP1 also cleared from blood more rapidly, indicating that permeability alone cannot explain capsid-dependent delivery [6].

The route of tubular access also matters. Vascular delivery can reach the tubular basolateral surface through peritubular and interstitial compartments, without passage through the urinary space [6]. Intravenous and renal-vascular delivery [16,17] approach renal barriers differently from pelvic or retrograde ureteral delivery [18,19]. Urinary administration begins in the urinary tract, but the administration site alone does not establish the cellular entry surface. Glomerular and peritubular endothelia also have distinct barrier properties [20]. Capsids influence tissue access, entry and intracellular processing, whereas promoters act after delivery to bias expression [21,22]. Neither a renal promoter nor local administration establishes kidney-exclusive vector distribution. Studies should relate histological injury and viable target-cell numbers to blood and urinary vector kinetics, cell-resolved expression and extra-renal exposure [5,6,11].

### 2.3. Duration and Platform Selection

The required duration follows the indication and cargo pharmacology, rather than the diagnostic category alone. A brief intervention may be sufficient to alter an early IRI pathway; sustained replacement may be needed for a continuously required structural protein. Temporary immune modulation could help during an early inflammatory window, but persistent alloimmune risk may require a different strategy. These are indication-specific design questions, not fixed rules assigning all native-kidney disease to durable therapy and all graft conditioning to transient therapy.

AAV may support persistent expression in suitable cells, although onset, turnover, inflammation and anti-vector immunity constrain its use and repeat dosing [22,23,24]. Integrating lentiviral short hairpin RNA (shRNA) or transposon systems can extend activity but introduce insertion-related risks and limited reversibility [13,25]. Nonviral small interfering RNA (siRNA) has demonstrated renal target knockdown [12,14]; its temporary activity depends on intracellular exposure and target turnover, and recovery kinetics need direct measurement. Messenger RNA (mRNA) offers temporary protein production, whereas shRNA duration depends on the vector expressing it. Genome editing can permanently change the sequence of an edited cell even after the editor disappears; persistence of corrected tissue function still depends on cell survival and coverage [11].

Efficiency and safety cannot be ranked universally across these platforms. Viral vectors require attention to capsid immunity and persistent cargo activity; nonviral systems require effective cellular uptake, endosomal escape and tolerable repeat exposure. Lipid nanoparticle (LNP) formulations developed using selective organ targeting (SORT) have demonstrated siRNA-mediated renal target knockdown after systemic administration, although the tested formulation was predominantly lung-directed [26]. That evidence does not establish kidney-disease rescue or validate mRNA/LNP treatment during donor-kidney perfusion. Supplementary Table S1 compares renal evidence, specificity, duration, immunogenicity, safety and practical feasibility without treating unmatched experiments as head-to-head comparisons.

## 3. Genetic Therapeutics for Native Kidney Disease

### 3.1. Podocyte-Directed Therapies: Replacement and Secreted Complement Regulation

Podocin replacement addresses a cell-intrinsic defect in the slit-diaphragm apparatus. Ding et al. demonstrated benefit in genetic nephrotic-syndrome models using AAV and a nephrin promoter, including treatment after proteinuria developed [5]. This mechanism makes gene addition a plausible choice for an *NPHS2* loss-of-function disorder when viable podocytes remain and an appropriately expressed replacement protein can restore their function. Because podocin is not a secreted therapeutic, the construct must reach affected podocytes and restore appropriate protein localization; expression elsewhere cannot be assumed to provide cross-correction. Coverage, protein localization and albuminuria therefore need to be evaluated together. Existing scarring and podocyte depletion may limit recoverable benefit even when surviving cells are transduced.

PS-002 is designed to use podocytes for a different purpose: production of secreted complement factor I (CFI) to modify complement-mediated injury in immunoglobulin A (IgA) nephropathy, with bilateral renal-arterial administration described in its first-in-human phase 1/2 study (ClinicalTrials.gov: NCT07182227; UK Health Research Authority research summary: IRAS1011740). When checked on 5 October 2026, the registry listed the study as recruiting, with no posted results. CFI is a cofactor-dependent protease that cleaves C3b/C4b [27].

Complement-directed therapy has independent clinical support: systemic factor B inhibition with iptacopan slowed kidney-function decline in the randomized APPLAUSE-IgAN trial [28]. This supports the pathway rationale, rather than demonstrating CFI gene-transfer efficacy. For PS-002, the mechanistic rationale is control of a pathogenic extracellular pathway, rather than correction of a podocyte structural mutation. A secreted regulator could act beyond the cells producing it, so benefit may depend on local protein exposure and complement engagement more than on direct correction of every injured cell. This possible advantage remains to be established for PS-002 and creates a corresponding trade-off: circulating CFI exposure and infection-related safety require assessment. These approaches therefore suit different mechanisms of disease despite sharing a producer or target cell; clinical development of PS-002 does not establish human efficacy or superiority to structural replacement.

### 3.2. Proximal-Tubule Delivery and Inherited Tubular Disorders

For inherited proximal-tubule disorders, vector selection should start with the required molecular function and nephron-segment coverage. A membrane transporter must be expressed and correctly localized in the relevant epithelial cells; bulk renal expression is therefore an inadequate surrogate for restored reabsorption. Where that requirement is met, route and capsid can be chosen according to the dominant delivery constraint. In Furusho et al., locally delivered AAV-KP1 showed greater renal retention and less systemic escape than AAV9, supporting evaluation of that configuration when regional exposure is feasible [6]. Its performance does not establish the best capsid for every tubular disorder, and local delivery introduces procedural and distribution constraints.

Capsid evolution and genome design address other constraints. Rosales et al. developed AAV.k13/k20 through cross-species cycling and demonstrated renal expression in several experimental systems, including pig autotransplants [7]. Such evidence supports further assessment across species and delivery settings; it does not demonstrate correction of an inherited tubular disease, and the relevant capsid entry and post-entry mechanisms remain incompletely defined. Peek et al. showed efficient proximal-tubule reporter expression with self-complementary AAV.cc47, biased toward SGLT2-positive convoluted proximal tubules, and demonstrated split-vector reconstitution [8]. This segment preference makes coverage of the disease-relevant epithelial population an explicit selection criterion. A self-complementary genome bypasses second-strand synthesis and can be attractive for a compact cassette when post-entry expression is limiting, but its smaller packaging capacity constrains cargo and regulatory sequences. A split configuration expands the possible cargo while requiring both components to reach and reconstitute in the same cell [8,22]. Thus, stronger reporter expression is not sufficient to select a configuration unless the therapeutic cassette fits and the required epithelial population is reached.

Garcia et al.’s preprint further emphasized cassette and administration effects; its short human-kidney experiment demonstrated vector DNA delivery and retention, without independently establishing transgene protein expression or a transplant outcome [9]. Woodard et al. provided a disease-specific test: renal-pelvic transposon delivery of *Slc3a1* partially reduced urinary cystine in cystinuric mice, without a significant reduction in stone formation [25]. That result links transporter replacement to biochemical activity while leaving the coverage needed to prevent stones unresolved. Integration offers potential persistence but adds insertion-related risk, and hydrodynamic delivery requires assessment of pressure injury and clinical feasibility. Candidate selection therefore requires matched therapeutic-cargo comparisons and a functional endpoint appropriate to the disorder.

### 3.3. Cystic Kidney Disease: Pathway Modulation and Sequence Correction

Pathway modulation and sequence correction address different biological levels in autosomal dominant polycystic kidney disease (ADPKD). Ciobanu and Cebotaru selected AAV1 partly because cystic epithelia showed altered distributions of sialylated glycans associated with AAV1 attachment, providing a mechanistic rationale for testing this capsid [29]. The observed cyst transduction does not establish that altered glycosylation was its sole cause or that AAV1 is superior to engineered renal capsids. AAV1-delivered Δ27–264 cystic fibrosis transmembrane conductance regulator (CFTR) reduced cyst size, cyst area and epithelial proliferation in the tested *Pkd1* model. The truncated protein does not itself conduct chloride; it can modify processing and trafficking of endogenous CFTR, and increased basolateral CFTR localization was observed. Altered transport and proliferative signaling are therefore candidate mediators of benefit, but their causal contributions and a renal functional benefit were not established [29].

A pathway-directed cargo could be suitable when the relevant downstream abnormality is shared across variants, although broader genotype coverage remains a hypothesis for this construct and continued activity may be required. Sequence correction instead requires a pathogenic variant compatible with the editor and guide. Cheng et al. used dual-AAV9 adenine base editing to correct a defined *Pkd1* variant and improve disease outcomes in humanized mice [11]. For a compatible variant, this directly addresses the causal sequence and can produce a lasting change in successfully edited cells. Its trade-offs include component co-delivery, incomplete cellular coverage and unintended editing; efficacy in one variant does not establish general applicability to ADPKD. The cadherin-16/Ksp promoter increased renal-tubular editing relative to the comparator promoter, but hepatic reporter expression and partial restoration of hepatic polycystin-1 remained detectable [11]. The studies therefore support choosing the molecular strategy by genotype, target-cell access and required duration, rather than ranking AAV1 against AAV9 from unmatched experiments.

### 3.4. Glomerular Endothelial Immunomodulation in Autoimmune Disease

Wu et al. engineered AAV2-GEC to deliver the immunoglobulin G (IgG)-degrading enzyme of *Streptococcus pyogenes* (IdeS) to glomerular endothelial cells in anti-glomerular basement membrane (anti-GBM) disease [10]. IgG cleavage reduced glomerular antibody-associated injury and albuminuria in mice. Human primary glomerular endothelial cells also showed enhanced transduction relative to wild-type AAV2; these in vitro results do not establish human therapeutic efficacy. Longer reporter or enzyme-expression follow-up in separate cohorts does not define the duration of disease benefit. Circulating IdeS demonstrates that the location of production alone cannot define the extent of IgG cleavage.

## 4. Kidney Transplantation as a Gene-Transfer Platform

**Figure 2:**
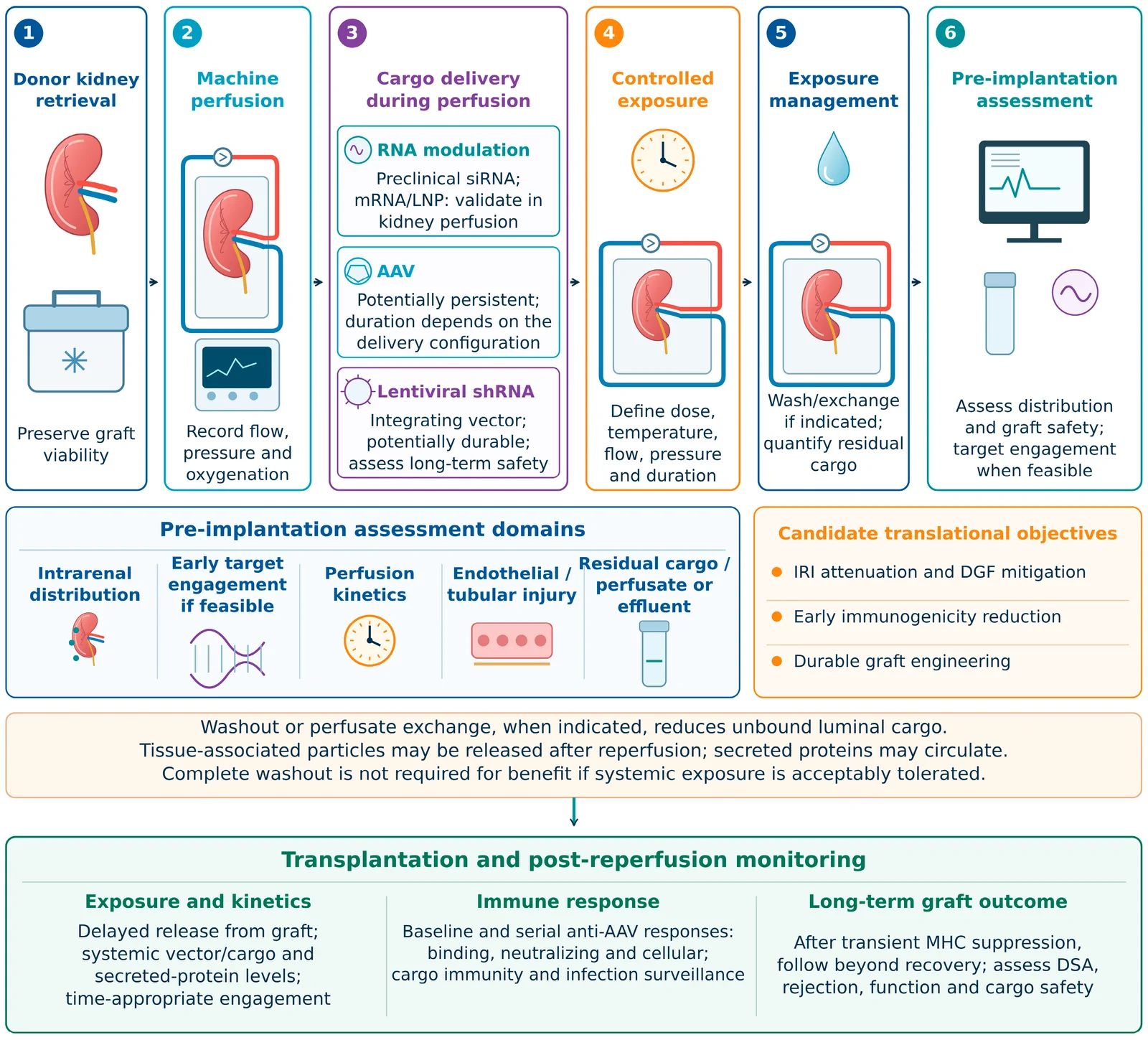
**Ex vivo donor-kidney gene transfer, graft assessment and recipient surveillance.** Controlled ex vivo delivery is followed by graft assessment and recipient surveillance. Washing or perfusate exchange is used when indicated; complete washout is not mandatory. Removal of unbound luminal cargo cannot exclude delayed tissue-associated release or systemic secretion of therapeutic proteins. Assess graft benefit alongside procedural and systemic tolerability against an appropriate comparator. Target engagement may require post-implantation assessment when expression or knockdown is delayed. Recipient monitoring includes exposure, anti-vector/cargo immunity, infection and graft outcomes; follow transient MHC suppression beyond recovery. Abbreviations: AAV, adeno-associated virus; DGF, delayed graft function; DSA, donor-specific antibody; IRI, ischemia–reperfusion injury; LNP, lipid nanoparticle; mRNA, messenger RNA; MHC, major histocompatibility complex; shRNA, short hairpin RNA; siRNA, small interfering RNA.

Donor-kidney treatment creates an opportunity to intervene before recipient exposure. Clinical studies support the feasibility of normothermic machine perfusion (NMP) for organ preservation and assessment [30,31]; added gene-transfer interventions require separate efficacy validation [32]. Recirculation and controlled exposure can improve contact with cargo, yet intrarenal distribution may remain uneven, particularly in injured microvasculature. Available treatment time, temperature-dependent uptake and expression, perfusion-related injury and integration into procurement and implantation schedules constrain delivery. Residual tissue-associated cargo can also reach the recipient after reperfusion. Figure 2 links the controllable ex vivo phase to the surveillance required after implantation.

### 4.1. Transient Graft Conditioning with RNA

The therapeutic purpose of transient RNA depends on the pathway being modified. Zheng et al. used a *C3/RelB/Fas* siRNA cocktail during donor-kidney perfusion and preservation to attenuate IRI in syngeneic transplantation [12]. Lin et al. used modified siRNA targeting beta-2-microglobulin (*B2m*) and the class II major histocompatibility complex transactivator (*Ciita*) during NMP and reported early benefit in renal allografts [14]. These interventions address acute tissue injury and antigen presentation, respectively; their results should not be treated as interchangeable evidence of rejection prevention.

For transient MHC suppression, early protection could permit a lasting state of graft protection, including accommodation, or simply postpone alloimmune priming. These alternatives remain hypotheses; neither accommodation nor tolerance was established by the short-term observations. Experiments should use matched conventional immunosuppression and continue beyond measured MHC recovery, with serial graft expression, biopsy, donor-specific antibody (DSA), rejection and survival assessments. Effects on natural killer (NK) recognition after MHC I reduction also require testing.

Complete washout is not an absolute prerequisite for therapeutic usefulness, and zero off-target exposure is not the appropriate universal goal. Washing or perfusate exchange should be selected according to the cargo, delivery process and exposure profile. A meaningful reduction in graft injury or acute rejection may justify limited systemic exposure or activity if its adverse effects are tolerable in comparison with standard systemic immunosuppression. This benefit–risk assessment requires measurement of recipient exposure and relevant adverse outcomes after reperfusion; an efficient wash alone cannot establish tolerability.

### 4.2. Durable Allograft Engineering with Viral Vectors

Yuzefovych et al. demonstrated sustained *B2m*/*Ciita* suppression after lentiviral shRNA delivery to perfused rat kidneys and subsequent allotransplantation [13]. The six-week molecular findings establish durable target engagement, rather than rejection prevention or tolerance. Integrating silencing could maintain reduced antigen presentation, but persistent effects on infection surveillance, NK responses and insertion-related safety may limit its advantages over a reversible intervention.

Tomasoni et al. demonstrated AAV9-mediated expression of LEA29Y, a modified cytotoxic T-lymphocyte-associated protein 4–immunoglobulin fusion protein, in non-human-primate autotransplanted kidneys [33]. Although serum protein was below detection, the primary study also showed hepatic LEA29Y transcript signal. These observations support renal expression feasibility while demonstrating that undetectable serum protein cannot establish graft-exclusive expression. Autotransplantation cannot demonstrate protection from an allogeneic response. LEA29Y expression and sustained MHC silencing therefore provide different forms of molecular evidence; rejection protection requires cargo-specific testing in allotransplantation.

### 4.3. Pre-Implantation Assessment and Perfusion Biology

The treatment process should specify vector or RNA dose, exposure time, temperature, flow, pressure and the strategy used to manage residual cargo, including washing or perfusate exchange when indicated. Pre-implantation assessment can examine distribution across cortex and medulla, perfusion kinetics, injury and residual free cargo. Immediate target engagement is useful when kinetically feasible, but AAV expression and RNAi-mediated protein reduction may require assessment after implantation. Extending perfusion solely to obtain an early expression signal could increase injury without improving the therapeutic configuration.

Human-kidney studies identified stress-related cellular responses [34] and inflammatory transcriptional responses [35] during NMP. During prolonged red-cell-based perfusion, hemolysis, iron accumulation and ferroptosis-associated changes have also been reported [36]. These findings support protocol optimization and suitable perfusion-only controls; they do not imply that every perfusion regimen produces the same injury. Cargo-induced effects must be separated from preservation injury and baseline donor damage. Translation requires reproducible regional delivery within a clinically workable treatment interval, rather than simply demonstrating activity after prolonged laboratory exposure.

### 4.4. Complementary In Situ and Recipient-Directed Strategies

Earlier work illustrates delivery configurations beyond machine perfusion. Donor-kidney Fas ligand transfer in situ and recipient-directed AAV-mediated interleukin-10 expression both prolonged allograft survival in rat models [37,38]. The former modifies the graft, and the latter uses an extra-renal site to influence transplantation systemically. These studies support immunomodulatory mechanisms while carrying different exposure profiles and clinical workflows. Recipient-directed benefit should therefore be distinguished from kidney-targeted delivery.

### 4.5. Proposed Genome Editing and Antibody-Directed Graft Strategies

Permanent ex vivo editing could modify donor-cell pathways without sustained editor expression, but sufficient coverage, unintended sequence changes and long-term graft safety must be established. Permanent loss of immune-recognition pathways may also be less reversible than temporary silencing. Such configurations remain proposals until direct renal-allograft experiments establish molecular modification and functional benefit.

Clinical IgG cleavage provides an independent alloimmune rationale for considering graft-directed IdeS. Systemic imlifidase enabled transplantation in highly sensitized recipients by cleaving preformed IgG antibodies, although post-transplant ABMR still occurred [39]. In a randomized trial of established ABMR, Halleck et al. found a larger early reduction in DSA with imlifidase than with plasma exchange: the median maximum reduction in summed DSA within the first 5 days was 97% versus 42%. DSA rebounded around days 6–12, and the trial did not demonstrate clinical benefit over 6 months [40]. Thus, clinical IgG cleavage can rapidly modify alloantibody exposure, but antibody reduction alone does not establish sustained graft protection.

Separately, Wu et al. demonstrated renal delivery and IgG-cleaving activity in experimental anti-GBM nephritis [10]. This delivery evidence, combined with the independent clinical imlifidase rationale, motivates a new hypothesis: controlled IdeS production in the graft might reduce DSA-mediated microvascular injury. It does not transfer autoimmune efficacy to alloimmune disease. Anti-GBM antibodies and DSA recognize different targets; alloimmune injury involves glomerular and peritubular compartments, and DSA production can continue. Direct allograft testing must establish adequate microvascular exposure and benefit under a clinically relevant immunosuppressive regimen.

Any advantage of local production must be balanced against expression delay and limits on stopping persistent activity. Ex vivo AAV delivery cannot be assumed to provide enough IdeS to protect against preformed DSA immediately after reperfusion. Clinical IdeS studies showed anti-enzyme antibody responses and recovery of IgG after cleavage [41]; these findings cannot define the kinetics or safety of persistent graft expression. IgG cleavage can also impair therapeutic-antibody function, as demonstrated experimentally for rituximab [42], and clinical consensus guidance specifies timing constraints for accompanying IgG-based treatments [43]. Prolonged graft expression could compromise protective IgG, so infection risk requires direct testing. Graft expression does not guarantee local pharmacology because secreted IdeS may circulate. Direct allograft experiments should relate expression onset and microvascular coverage to intact and cleaved DSA, tissue injury, systemic IgG function, anti-IdeS immunity and treatment compatibility. A systemic-enzyme comparator should test whether graft-directed production offers additional benefit. Regulated or time-limited expression is a rational design option whose advantage remains to be demonstrated.

Table 1 summarizes experimental evidence in native-kidney disease and donor-kidney engineering, including delivery configurations, doses, observation windows, outcomes and safety findings. It distinguishes delivery or molecular activity from disease benefit.

## 5. Safety and Translational Evaluation Across Kidney Gene-Transfer Strategies

### 5.1. Platform- and Cargo-Specific Safety

Safety assessment must reflect both the delivery platform and the intended biological effect. AAV programs require evaluation of capsid- and cargo-directed immunity, systemic exposure, inflammation and persistence [22,23,24]. Integrating vectors and transposons require insertion-site and clonal surveillance; editing programs require unintended-edit analysis and follow-up appropriate to irreversible sequence changes [11,13,25]. A negative candidate-site assay is reassuring within its tested scope but cannot exclude every rare genomic event. Persistent immune modulation further requires assessment of infection, immune surveillance and the ability to stop or reverse harmful activity.

Human kidney organoids can reveal cell responses that animal experiments may miss; Gupta et al. identified an AAV2-associated nuclear factor κB (NFκB)-mediated nephrotoxic response in organoids [44]. However, static organoids are immature, lack normal continuous microvascular perfusion and do not reproduce physiological hydrostatic filtration pressure or whole-body clearance. Flow-enhanced systems improve vascularization and maturation but do not recreate every feature of an adult filtering kidney [45]. Organoids are therefore complementary to perfused human kidneys and in vivo models, particularly for cellular toxicity, rather than sufficient models of shear-dependent access or recipient pharmacokinetics.

### 5.2. Vascular Spillover and Systemic Exposure

Local delivery can improve renal transduction and reduce extra-renal exposure in selected configurations [6,17], while still permitting vascular escape. After renal-arterial administration, the renal vein provides a route to the systemic circulation. A renal-arterial route is consequently not synonymous with recipient containment. Studies should quantify circulating vector or cargo during delivery and after release, assess extra-renal distribution and relate exposure to liver injury, complement activation and other platform-relevant adverse effects. Secreted therapeutic proteins require separate pharmacokinetic measurement even when vector distribution is predominantly renal.

Temporary vascular isolation or balloon occlusion may increase residence time and reduce immediate escape, but the renal evidence does not establish that these techniques are universally required. Their benefit must be tested against altered flow, venous congestion, ischemia and procedural injury. Comparisons should match renal target engagement where possible: a lower systemic signal at the cost of inadequate delivery or damaged tissue does not establish a superior treatment. The clinically relevant decision is whether the route produces sufficient renal benefit with acceptable procedural and systemic risk.

### 5.3. Exposure Management and Post-Transplant Surveillance

When used, ex vivo washing primarily removes unbound cargo from the vascular lumen and perfusate. It cannot establish removal of matrix-bound or cell-associated particles that may be released after reperfusion. Low final-effluent vector concentration is therefore an initial exposure measure, not proof of absent recipient biodistribution. The need for washing or perfusate exchange is strategy-dependent; usefulness rests on graft benefit and tolerable procedural and systemic effects rather than complete removal of every residual particle.

The AAV observations must be interpreted at the level actually measured. Tomasoni et al. screened pre-existing capsid antibodies in 16 non-human-primate sera to select AAV9. After autotransplantation, renal LEA29Y expression and hepatic transcript signal were detected, whereas serum protein was below detection [33]. The hepatic signal is evidence of extra-graft expression; protein non-detection cannot quantify vector spillover or exclude systemic exposure. Pre-treatment antibody screening is also distinct from serial recipient immune surveillance. A complete quantitative recipient vector profile together with baseline and serial capsid-binding, neutralizing and cellular responses could not be verified for the cited ex vivo AAV configurations. The separate lentiviral study by Yuzefovych et al. found graft-only vector PCR in sampled tissues at week 6 [13]; that late result cannot exclude an earlier systemic pulse and does not provide AAV-specific immunity evidence.

Prospective surveillance should quantify serial perfusate or effluent concentrations and released mass during any washing or exchange, then measure recipient plasma vector or RNA soon after reperfusion and over subsequent sampling intervals. Suitable preclinical models should include extra-graft tissue vector analysis and relevant shedding assays. Secreted-cargo concentrations and biological activity require separate pharmacokinetic assessment. Measurements should report compartments, units, sampling times and detection limits. For ex vivo AAV, paired baseline and serial capsid-binding and neutralizing antibodies, together with capsid-specific cellular assays, should be related to exposure, graft inflammation and expression persistence [23,24]. Liver injury, complement activation, infection and cargo-specific effects—including total and functional IgG where relevant—should be evaluated against the treatment comparator. Unverified measurements remain evidence gaps, not negative safety findings. Where existing data are insufficient, prospective experiments should provide the missing measurements; this review cannot generate new quantitative data.

### 5.4. Model Selection and Evidence Comparability

Species, age, disease activity, cargo, promoter, genome configuration, route and timing all affect apparent delivery efficiency. Reporter studies, human-organ exposure experiments, autotransplants and allotransplants answer different questions. Large-animal feasibility narrows a delivery gap but does not replace a disease endpoint, and a single discarded human kidney cannot establish reproducibility or recipient safety. Table 1 therefore reports study-specific outcomes without ranking delivery efficiency across experiments.

The effective fraction of corrected cells is an empirical scientific question. Studies should measure regional and cell-type coverage together with dose, target engagement and functional outcomes, estimating the exposure–response relationship and its uncertainty. A universal minimum fraction should not be imposed before those data exist. Follow-up must distinguish expression persistence, molecular action and sustained therapeutic benefit, including late rejection after transient immune effects recover.

## 6. Decisive Experiments and Clinical Development

Clinical development requires experiments that connect delivery and target-cell coverage with molecular activity, functional benefit and safety. Table 2 summarizes candidate clinical configurations, their principal uncertainties and the experiments and measurements needed to evaluate them.

For native disease, the next experiment should link dose to renal and extra-renal distribution, relevant cell coverage, molecular engagement and functional benefit. For graft engineering, that sequence must include perfusion controls, clinically feasible exposure and post-reperfusion surveillance. Human-relevant models should test whether the dose and treatment interval can be achieved without compromising organ quality or the implantation workflow [32].

Quantitative decisions should use predefined endpoints and indication-specific benefit and tolerability margins, informed by natural history and standard care. Table 2 specifies exposure, cell coverage, molecular and disease endpoints, including urinary albumin-to-creatinine ratio and glomerular filtration rate (GFR), for each indication. Distribution assays should report tissue compartment, time, detection limits and normalization. For an RNA intervention, the time to recovery of target expression should be measured and follow-up should extend beyond it. For a secreted immunomodulator, systemic concentration and biological activity should be related to both graft effect and adverse outcomes.

The available renal studies do not support common numerical go/no-go cutoffs across indications. Effective coverage and acceptable exposure should be estimated in dose–response experiments, followed by prospectively justified criteria for the next study. A candidate should advance when benefit is reproducible against an appropriate comparator and uncertainty about procedure, exposure, immunity and persistent activity has been reduced sufficiently for the proposed clinical use. Early target engagement without disease benefit remains a valuable intermediate result but should be reported at that level.

## 7. Conclusions

Kidney-targeted gene transfer is developing through disease-specific native-kidney treatment and pre-implantation donor-kidney engineering. Progress depends on connecting renal delivery to cell-resolved activity and a clinically relevant outcome. Capsid engineering, promoter selection and route optimization are complementary tools whose performance remains dependent on context. For transplantation, the next gains require more uniform intrarenal delivery, exposure intervals compatible with clinical practice and control of perfusion injury. Strategy-dependent management of residual cargo must be evaluated together with delayed release, recipient immunity and tolerability; complete washout is not a universal prerequisite. Transient MHC suppression requires follow-up after expression recovers, and graft-directed IdeS remains a hypothesis requiring direct alloimmune testing. Reproducible dose–response relationships and proportionate long-term surveillance will determine which configurations can move from encouraging preclinical results to clinical benefit.

## Figures and Tables

**Table 1:** Study-level evidence for kidney-targeted genetic therapeutics.

Study and Model	Delivery Configuration and Dose	Observation Window	Demonstrated Outcome and Category	Safety Findings and Evidence Boundary
Ding et al. [5] *Nphs2*-deficient mice	AAV2/9–*NPHS1*–podocin; IV, 1.5 × 10^12^ vg/mouse. Knockout: before induction; knock-in: day 14 after induction, with proteinuria.	Expression: 8 weeks (knockout). Outcomes: 42 days after dosing (knock-in); separate survival follow-up to day 365 after induction.	Therapeutic preclinical evidence: albuminuria and disease phenotypes improved; survival prolonged in the knockout model.	Liver/spleen transgene transcripts detected with the minimal human nephrin promoter. Survival follow-up does not establish equally long expression.
Furusho et al. [6] Healthy/CKD mice; NHPs	Reporter AAV-KP1/AAV9. Healthy mice: IV, renal vein or pelvis, 3 × 10^11^ vg/mouse. CKD IV: 10^13^ vg/kg. NHP renal-pelvic delivery: KP1, 6.3 × 10^12^–1.7 × 10^13^ vg/animal; AAV9, 2.4–2.7 × 10^13^ vg/animal; 10-min dwell.	Individual mouse validation: 2 weeks. NHP: 3 weeks.	Reporter evidence: route-, species- and disease-dependent transduction. Local KP1 reached proximal tubules; CKD altered AAV9 access to podocytes/tubules.	Blood vector kinetics and liver biodistribution assessed in mice and NHPs. NHPs had transient liver-enzyme increases; transduced regions in four animals showed mononuclear infiltration/tubulitis. No disease rescue tested.
Rosales et al. [7] Mice; pig autotransplants	Reporter k13/k20 IV: 5 × 10^13^ vg/kg in mice. Pig: arterial + ureteral delivery, 5.9 × 10^12^ vg/route (k13, cold storage) or 6.1 × 10^12^ vg/route (k20, perfusion); 2 h exposure.	Mouse: ~30 days. Pig: 14 days (k13); 9 days (k20).	Reporter evidence: predominantly proximal-tubule expression across models. Separate mouse PD-L1 experiment established protein expression, without a rejection-efficacy test.	Mouse liver vector genomes/expression persisted. Pig autotransplantation does not test allorejection; durability and therapeutic safety require direct study.
Peek et al. [8] Mice	scAAV.cc47–CBh–mCherry IV: 5 × 10^13^ vg/kg; dose series 10^10^–10^12^ vg/mouse. Split reporter: 10^11^ vg for each half.	~30 days (capsid comparison); 3 weeks (dose series/split reporter).	Reporter evidence: strong proximal-tubule transduction; split-vector reconstitution demonstrated. Expression improvement did not imply similarly improved vector biodistribution.	Liver and heart expression observed. No renal disease-rescue experiment; long-term therapeutic safety and human efficacy not established.
Garcia et al. [9] Mice; one human kidney Preprint	Mouse ssAAV reporter IV: 10^11^ vg/mouse in the principal study. Human NMP: ssAAV9–GFP + ssAAV.cc47–mCherry, 10^13^ vg each; added at perfusion hour 2.	Mouse: 21 days. Human: 50 h perfusion, 48 h after vector addition.	Reporter optimization in mice. Human qPCR/PCR and DNA-targeted in situ assays support vector delivery/retention; they do not independently establish transgene protein expression.	Mouse liver expression observed. Single human organ and short perfusion window; no transplant outcome, disease rescue or in vivo immune-safety assessment.
Woodard et al. [25] *Slc3a1*−/− cystinuria mice	EF1α–*Slc3a1* piggyBac transposon: 50 μg + 10 μg transposase plasmid in 100 μL; unilateral hydrodynamic renal-pelvis injection.	*Slc3a1* RNA: to 6 weeks. Urinary cystine: day 154.	Therapeutic preclinical evidence: urinary cystine/creatinine decreased by ~44% at day 154; stone formation/area did not decrease significantly.	No liver transgene RNA detected in the tested assay. Integration-site safety and comprehensive procedural toxicity were not established; biochemical correction was partial.
Ciobanu and Cebotaru [29] Pkd1RC/RC mice	AAV1–Δ27–264-CFTR; single intraperitoneal administration. Dose inconsistently specified in the source ‡.	~60 days after dosing (~3 months of age).	Therapeutic preclinical evidence: preferential cyst transduction; cyst area/size and epithelial proliferation decreased.	Structural benefit; renal functional improvement was not an assessed endpoint. Dedicated long-term toxicology, immunogenicity and systemic biodistribution remain unresolved.
Cheng et al. [11] Humanized ADPKD mice	Dual-AAV9 ABE9/guide RNA, IV; combined doses. Pkd1RC/RC: Cbh, 10^11^ or 10^12^ vg/mouse at PN14/28; Ksp, 10^12^ vg at PN14. Pkd1RC/null: 10^12^ vg at PN7 with either promoter.	Outcomes at age 3/6 months; separate lifespan study in Pkd1RC/null mice.	Therapeutic preclinical evidence: sequence correction, improved cyst/function endpoints and longer survival. Ksp enhanced renal-tubular editing.	Extra-renal activity persisted, including hepatic reporter expression and partial hepatic polycystin-1 restoration. Tested candidate off-target sites and RNA-editing assays were reassuring within their scope; they do not exclude all rare genomic events.
Wu et al. [10] Mouse anti-GBM nephritis	CMV–AAV2-GEC–secretory IdeS, IV: 10^13^ vg/kg, 14 days before or 1 day after disease induction. Separate expression cohorts: 5 × 10^12^ vg/kg.	Disease endpoints: induction days 7/22. Separate IdeS–Nluc and GFP expression: to 240/360 days.	Therapeutic preclinical evidence: albuminuria and glomerular IgG/Fc/complement deposition decreased in anti-GBM disease.	Tested liver indices were normal. Circulating IdeS persisted in a separate expression cohort; long expression follow-up does not establish equally long anti-GBM efficacy.
Zheng et al. [12] Syngeneic mouse transplant	*C3/RelB/Fas* siRNA cocktail; intra-arterial donor-kidney perfusion during retrieval and storage in siRNA-containing solution. Dose and exposure duration: NV.	Post-transplant renal injury and graft survival assessed; numerical follow-up: NV.	Therapeutic preclinical evidence: target knockdown; reduced biochemical dysfunction, apoptosis and histological/inflammatory injury; survival benefit.	Syngeneic IRI model. Quantitative systemic exposure and dedicated toxicity could not be verified from accessible primary material.
Lin et al. [14] Rat renal allograft model	Cholesterol-modified siB2m/siCiita during donor-kidney NMP. Dose and exposure duration: NV.	POD3 MHC expression; POD7 injury/immune assessments; complete survival follow-up: NV.	Therapeutic preclinical evidence: reduced MHC and early rejection/immune markers, with improved graft function and recipient survival.	Early alloimmune benefit; protection after MHC recovery and tolerance remain unestablished. Quantitative systemic exposure/toxicity: NV.
Yuzefovych et al. [13] Lew.1W→Lew rat allografts	Lentiviral shB2m/shCiita: 1.5 × 10^11^ particles (p24 assay); 2 h vector perfusion with gradual warming to 31–32 °C, followed by staged washing.	6 weeks after transplantation.	Preclinical target engagement: sustained reporter; *B2m*/*Ciita* transcripts decreased by ~70%. Rejection prevention and tolerance were not demonstrated.	No additional LDH/histological injury over perfusion controls; cytokine changes observed. Vector PCR was graft-only in sampled tissues at week 6; earlier exposure is not excluded.
Tomasoni et al. [33] NHP autotransplants	AAV9–LEA29Y; ex vivo incubation for 1 h at 4 °C before autotransplantation. NHP dose: NV.	NHP follow-up: NV; separate rat feasibility arm: 21 days.	Preclinical expression evidence: renal LEA29Y transcript/protein; serum protein below detection. Autotransplantation did not test allorejection protection.	Pre-existing anti-capsid antibodies screened in 16 NHP sera. Hepatic LEA29Y transcripts detected; serum protein below detection. Numerical detection limit and complete serial recipient vector/anti-capsid profiles could not be verified.

Table 1 is a study-level inventory of tested configurations and demonstrated findings. Doses and observation windows refer to selected experiments; differences in models, cargos, genomes, routes, assays and timing prevent direct numerical ranking. Reporter expression, molecular target engagement and disease benefit are distinct categories. Survival follow-up is not a measurement of expression duration. NV, a quantitative parameter could not be independently verified from the accessible primary material; this is an access limitation, not evidence that the original study omitted the parameter. Verified findings are retained without inferring unavailable numbers. Safety statements describe the assays reported and do not constitute comprehensive safety assessments. ‡ The source inconsistently reports 10^12^ vg per animal, 2 × 10^12^ particles/kg and 10^12^ vg/kg; the administered dose therefore cannot be resolved from the published text [29]. Abbreviations for Table 1: AAV, adeno-associated virus; sc/ss, self-complementary/single-stranded; AAV2-GEC, engineered glomerular endothelial cell-tropic AAV2; ABE9, adenine base editor 9; ADPKD, autosomal dominant polycystic kidney disease; anti-GBM, anti-glomerular basement membrane; *B2m*, beta-2-microglobulin; CFTR, cystic fibrosis transmembrane conductance regulator; *Ciita*, class II MHC transactivator; CKD, chronic kidney disease; IdeS, IgG-degrading enzyme of *Streptococcus pyogenes*; IgG, immunoglobulin G; IRI, ischemia–reperfusion injury; IV, intravenous; LEA29Y, modified cytotoxic T-lymphocyte-associated protein 4–immunoglobulin fusion protein; MHC, major histocompatibility complex; NHP, non-human primate; NMP, normothermic machine perfusion; PN/POD, postnatal/postoperative day; sh/siRNA, short hairpin/small interfering RNA; vg, vector genomes. The prefixes si/sh identify the targeted gene. Additional terms: C3, complement component 3; CBh/Cbh, hybrid chicken beta-actin promoter; CMV, cytomegalovirus promoter; DNA, deoxyribonucleic acid; EF1α, elongation factor 1 alpha promoter; Fc, crystallizable fragment of IgG; GFP, green fluorescent protein; Ksp, kidney-specific cadherin-16 promoter; LDH, lactate dehydrogenase; Nluc, NanoLuc luciferase; *NPHS1*/*NPHS2*, nephrin/podocin genes; PD-L1, programmed death ligand 1; PCR/qPCR, polymerase chain reaction/quantitative PCR; RNA, ribonucleic acid.

**Table 2:** Candidate clinical configurations and decisive validation for translation.

Clinical Objective	Target, Cargo, Route, Intended Duration and Evidence	Off-Target Exposure and Key Uncertainty	Decisive Next Experiment	Proposed Measurements and Outcomes
*NPHS2* replacement	Podocytes; *NPHS2*; systemic AAV; sustained replacement. Therapeutic preclinical evidence [5]. Local efficacy requires testing.	Systemic AAV can reach the liver; effective podocyte coverage and tolerable exposure remain uncertain.	Relate dose and podocyte coverage to function; compare systemic and local delivery at matched target engagement.	Renal/hepatic vg per diploid genome; transduced podocytes (%); podocin-positive slit diaphragms (% evaluated); UACR (mg/g), measured GFR and benefit duration.
IgAN complement regulation (PS-002)	Podocytes; AAV-mediated secretion of complement factor I (CFI); bilateral renal-arterial delivery; sustained activity sought. Registered phase 1/2 development (NCT07182227; source note below); human efficacy unestablished. CFI and complement-treatment rationale [27,28].	Secreted CFI may circulate; renal complement benefit must be related to systemic exposure and infection risk.	Early clinical dose–exposure–response assessment.	Plasma CFI (ng/mL); expressing podocytes (%) when measurable; complement activity (% baseline); proteinuria, eGFR slope and infection incidence.
Inherited proximal-tubule disorders	Affected proximal-tubule segment; disease-specific replacement; systemic or local delivery. AAV reporter evidence [6,7,8,9] and *Slc3a1* transposon biochemical benefit [25]. Duration depends on cargo/platform.	Vector/cargo distribution is formulation- and route-dependent; liver exposure and epithelial coverage require direct assessment.	Compare the same therapeutic cargo across matched configurations.	Vector genomes per diploid genome or cargo mass/g tissue; cargo-positive target cells (%); transporter activity (% control); urinary substrate excretion, stone burden and GFR.
ADPKD sequence correction	Tubular/cyst epithelium; dual-AAV9 ABE9/guide RNA; IV; lasting correction in edited cells. Therapeutic preclinical evidence [11].	Extra-renal AAV9 activity persists; co-delivery and unwanted edits remain concerns.	Link corrected-cell/allele fractions to disease response; follow genomic safety.	Renal/hepatic vg per diploid genome; target cells reached (%); corrected alleles/unintended edits (%); cyst volume fraction, GFR and surveillance duration.
Peri-transplant IRI/DGF	Graft parenchyma/endothelium; injury-targeting siRNA during perfusion; transient activity. Therapeutic preclinical IRI evidence [12].	Post-reperfusion RNA exposure requires measurement; acute injury benefit does not establish alloimmune protection.	Large-animal transplantation with active-siRNA, control-siRNA and perfusion-only arms.	Effluent/plasma siRNA (ng/mL); target cells reached (%); target reduction (% control); GFR, injury score and survival; DGF frequency in subsequent clinical studies.
Reversible graft immunogenicity reduction	MHC-expressing graft cells; siB2m/siCiita during NMP; transient activity. Therapeutic preclinical evidence [14].	Post-reperfusion RNA exposure requires measurement; protection after MHC recovery is uncertain.	Follow beyond measured MHC recovery under matched immunosuppression; assess DSA/NK responses.	Plasma siRNA (ng/mL); target cells reached (%); MHC I/II (% control), recovery time; rejection, validated DSA assay, NK activity and survival.
Durable graft immune modulation	Selected graft compartments; ex vivo lentiviral shB2m/shCiita or AAV–LEA29Y; sustained activity sought. Molecular/expression evidence [13,33].	Tissue-associated vector may escape; integrating silencing and circulating cargo require separate safety assessment.	Compare durable/reversible configurations in allotransplantation with long-term surveillance.	Extra-graft vector, plasma LEA29Y (ng/mL); expressing cells (%); MHC reduction (% control) or functional costimulatory-pathway blockade; rejection, survival, infections and tumors.
Proposed ABMR application of IgG cleavage	Graft microvascular endothelium; controlled IdeS expression; route and duration unvalidated. Proposed use; independent systemic imlifidase rationale [39,40].	Delayed onset, systemic IgG cleavage, anti-IdeS immunity and IgG-treatment interactions; stopping activity and microvascular coverage require validation.	Test controlled IdeS in alloantibody-mediated graft injury under matched immunosuppression, with systemic-enzyme and vector-only comparators.	Circulating IdeS (ng/mL); expressing microvascular cells (%); intact/cleaved IgG and DSA; microvascular injury, graft function/survival, infections and treatment compatibility.

Table 2 describes candidate clinical designs and proposed validation, rather than the measured dose, follow-up or outcome history of individual studies. Maturity denotes the highest outcome demonstrated in the relevant configuration: reporter/molecular evidence, therapeutic preclinical evidence, clinical development or proposed use. Clinical development does not establish human efficacy, and evidence is not inherited across species, cargos or routes. Proposed measurements cover exposure, relevant cell coverage, molecular engagement and functional benefit. Specify compartment, timing, assay, normalization and detection limits. These are measurable endpoints, not universal numerical cutoffs: dose–response studies establish effective coverage and tolerable exposure; indication-specific progression margins require prospective justification. Use washing or perfusate exchange when indicated by the strategy; quantify residual cargo and delayed release after reperfusion. Allow sufficient time for expression or knockdown. Viral, integrating and editing configurations require appropriate immune, insertion-site and unintended-edit surveillance. After IdeS, distinguish intact and cleaved DSA with validated reagents; MFI alone can reflect cleavage-dependent detection and need not indicate intact Fc function [42]. Systemic imlifidase studies provide the alloimmune rationale [39,40], not evidence of vector-mediated ABMR efficacy. NK susceptibility after MHC I reduction remains a hypothesis. PS-002 sources: ClinicalTrials.gov, NCT07182227 (https://clinicaltrials.gov/study/NCT07182227, accessed on 5 October 2026), and the UK Health Research Authority research summary, IRAS1011740, REC25/LO/0452 (https://www.hra.nhs.uk/planning-and-improving-research/application-summaries/research-summaries/phase-12-study-of-ps-002-a-gene-therapy-for-the-treatment-of-iga-nephropathy/, accessed on 5 October 2026). Checked 5 October 2026: the registry listed recruitment with no posted results. These official records support the planned trial configuration. References [27,28] support the CFI mechanism and IgAN complement-treatment rationale, respectively; neither establishes PS-002 efficacy. Abbreviations for Table 2: AAV, adeno-associated virus; ABE9, adenine base editor 9; ABMR, antibody-mediated rejection; ADPKD, autosomal dominant polycystic kidney disease; B2m, beta-2-microglobulin; CFI, complement factor I; Ciita, class II MHC transactivator; DGF, delayed graft function; DSA, donor-specific antibody; eGFR, estimated glomerular filtration rate; GFR, glomerular filtration rate; IdeS, IgG-degrading enzyme of Streptococcus pyogenes; IgAN, immunoglobulin A nephropathy; IgG, immunoglobulin G; IRI, ischemia–reperfusion injury; IV, intravenous; LEA29Y, modified cytotoxic T-lymphocyte-associated protein 4–immunoglobulin fusion protein; MFI, mean fluorescence intensity; MHC, major histocompatibility complex; NK, natural killer; NMP, normothermic machine perfusion; NPHS2, podocin gene; shRNA, short hairpin RNA; siRNA, small interfering RNA; UACR, urine albumin-to-creatinine ratio; vg, vector genomes. The prefixes si/sh before gene symbols identify the gene targeted by siRNA/shRNA.

## Data Availability

No new data were created or analyzed in this narrative review. Data sharing is not applicable to this article.

## Supplementary Materials

The following supporting information can be downloaded at: https://www.mdpi.com/article/10.3390/genes17101270/s1, Table S1. Viral and nonviral platforms in the renal evidence base.
