# Supplementary material for: Kidney-Targeted Gene Transfer: Therapeutic Advances in Native Kidney Disease and Emerging Ex Vivo Allograft Engineering
Source: Genes (Basel). 2026 Oct 13;17(10):1270. doi: 10.3390/genes17101270 (PMC13704283; doi:10.3390/genes17101270)
Supplement: Supplementary file 1 [file genes-17-01270-s001.zip › genes-4535338-supplementary.pdf]

Supplementary Material

Table S1. Viral and nonviral platforms in the renal evidence base.

Platform	Renal evidence and maturity	Efficiency and specificity	Duration, immunity and control	Safety and practical feasibility
AAV gene addition or editing	Preclinical disease benefit [5,10,11]; cyst-size reduction [29]; large-animal reporters [6,7]; human renal DNA delivery (preprint) [9]; registered PS-002 IgAN clinical development (NCT07182227).	Capsid/cassette/route dependent. Renal promoter bias is not exclusive biodistribution; dual vectors need co-delivery.	Persistent expression or sequence correction; cell turnover and anti-capsid immunity affect duration/redosing.	Capsid/cargo immunity, inflammation and systemic exposure; renal dose and route must be clinically feasible.
Lentiviral shRNA	Persistent *B2m*/*Ciita* suppression in rat allografts; rejection prevention unproven [13].	Controlled perfusion exposure; coverage and recipient distribution still require assessment.	Integration supports persistent shRNA expression with limited reversibility; immune response remains relevant.	Insertion sites, persistent immune suppression and inflammation; validated organ perfusion and manufacturing needed.
Nonviral siRNA	IRI attenuation [12] and early alloimmune benefit [14].	Intracellular delivery and target knockdown determine activity, not organ exposure alone.	Transient knockdown; follow beyond target recovery. Innate immune activation and repeat exposure require assessment.	Off-target silencing, RNA immune effects and MHC recovery; ex vivo exposure must fit treatment time.
siRNA lipid nanoparticles	IV SORT-mediated renal Tie2 knockdown; kidney-disease rescue unproven [26].	Formulation dependent; tested DOTAP-50 was predominantly lung-directed with renal activity.	Knockdown assessed through day 10 after two-dose treatment; chronic dosing and immune tolerability unestablished.	No significant acute chemistry changes or overt histological injury at 24 h after 1 mg/kg; siTie2 increased lung vascular permeability. Chronic tolerability remains unresolved.
Nonviral mRNA / LNP (proposed graft use)	Temporary protein production is a candidate strategy. The cited renal LNP study demonstrates siRNA target knockdown [26]; it does not establish donor-kidney mRNA/LNP therapeutic efficacy.	Requires renal cellular uptake and endosomal escape.	Transient protein production; formulation/cargo affect onset and duration. Innate immune and repeat-dose effects require testing.	Perfusion injury, lipid/RNA immunity and systemic cargo release; manufacturing and clinically feasible exposure require validation.

Plasmid–transposon	Partial urinary-cystine correction; no significant stone reduction [25].	Patchy proximal-tubule expression after hydrodynamic pelvic delivery.	Integration may support persistence; repeat delivery and long-term clonal effects unestablished.	Insertion sites and pressure injury; invasive hydrodynamic delivery requires human-route validation.

The comparison concerns the renal configurations cited in the main manuscript. No universal numerical ranking of efficiency, immunogenicity or safety is supported by unmatched models. Genome editing is an effect mechanism and may use different delivery platforms; sequence correction, persistent expression and integrating silencing differ in reversibility.

The siRNA-LNP row concerns systemic renal target engagement, not donor-kidney mRNA/LNP efficacy. Clinical development does not establish human benefit. Indication-specific dose–response and safety comparisons are needed before platform preference can be established.

References use the numbering of the main manuscript. AAV, adeno-associated virus; CFI, complement factor I; IgAN, immunoglobulin A nephropathy; *B2m*, beta-2-microglobulin; *Ciita*, class II major histocompatibility complex transactivator; IRI, ischemia–reperfusion injury; LNP, lipid nanoparticle; MHC, major histocompatibility complex; mRNA, messenger RNA; shRNA, short hairpin RNA; siRNA, small interfering RNA; SORT, selective organ targeting. DOTAP, 1,2-dioleoyl-3-trimethylammonium-propane. PS-002 design and status are attributed to ClinicalTrials.gov (NCT07182227; https://clinicaltrials.gov/study/NCT07182227) and the UK Health Research Authority research summary (IRAS1011740; REC25/LO/0452; https://www.hra.nhs.uk/planning-and-improving-research/application-summaries/research-summaries/phase-12-study-of-ps-002-a-gene-therapy-for-the-treatment-of-iga-nephropathy/), checked 5 October 2026. These official study records are distinct from the numbered literature references.
